# Stem Cell Therapy in Bladder Dysfunction: Where Are We? And Where Do We Have to Go?

**DOI:** 10.1155/2013/930713

**Published:** 2013-09-16

**Authors:** Jae Heon Kim, Sang-Rae Lee, Yun Seob Song, Hong Jun Lee

**Affiliations:** ^1^Department of Urology, Soonchunhyang School of Medicine, Seoul 140-743, Republic of Korea; ^2^National Primate Research Center, Korea Research Institute of Bioscience and Biotechnology, Ochang 363-883, Republic of Korea; ^3^Medical Research Institute, Chung-Ang School of Medicine, Seoul 156-756, Republic of Korea

## Abstract

To date, stem cell therapy for the bladder has been conducted mainly on an experimental basis in the areas of bladder dysfunction. The therapeutic efficacy of stem cells was originally thought to be derived from their ability to differentiate into various cell types. Studies about stem cell therapy for bladder dysfunction have been limited to an experimental basis and have been less focused than bladder regeneration. Bladder dysfunction was listed in MESH as “urinary bladder neck obstruction”, “urinary bladder, overactive”, and “urinary bladder, neurogenic”. Using those keywords, several articles were searched and studied. The bladder dysfunction model includes bladder outlet obstruction, cryoinjured, diabetes, ischemia, and spinal cord injury. Adipose derived stem cells (ADSCs), bone marrow stem cells (BMSCs), and skeletal muscle derived stem cells (SkMSCs) are used for transplantation to treat bladder dysfunction. The main mechanisms of stem cells to reconstitute or restore bladder dysfunction are migration, differentiation, and paracrine effects. The aim of this study is to review the stem cell therapy for bladder dysfunction and to provide the status of stem cell therapy for bladder dysfunction.

## 1. Introduction

Although numerous treatments for bladder dysfunction including bladder overactivity or underactivity have previously been developed, the improvement in voiding dysfunction is not been fully achieved. Stem cells are defined as cells with an ability to propagate themselves through self-renewal and generate mature cells of multiple lineages through differentiation [1]. Given their unique abilities of site, specific migration, plasticity and potential for tissue repair or regeneration, stem cells and their relationship to repair injury or damage in various organ systems have recent interest.

For the bladder dysfunction, bladder outlet obstruction (BOO) is a well-known and well-established bladder dysfunction model. Other bladder dysfunction models are still at an immature state. Medical and surgical efforts to treat and prevent BOO are ongoing, as are studies to better understand the effects and clear mechanisms of BOO at a cellular level.

Mesenchymal stem cells (MSCs) augment healing through cell replacement and stimulation of cell proliferation and angiogenesis. While numerous reports have shown the ability of MSCs to engraft tissues such as lung, liver, heart, and brain, data is still scarce about the repair of bladder dysfunction [2–4].

 The aim of this review is to provide the current status of stem cell therapy for bladder dysfunction and also to discuss future prospective on this issue. 

## 2. Stem Cells for Treatment of Bladder Dysfunction 

 MSCs are self-renewing cells with pluripotent capacity to differentiate into various cell types including osteoblasts, chondrocytes, myocytes, adipocytes, and neurons [5].

While all MSCs including bone marrow-derived stem cells (BM-MSCs), skeletal muscle-derived stem cells (SkMSCs), and adipose tissue-derived stem cells (ADSCs) exhibit similar biological properties and therapeutic capabilities, their availability and scalability differ greatly according to therapeutic purpose. For example, while SkMSCs require a long expansion time with complicated isolation procedure, ADSCs can be prepared within hours. 

 ADSCs are mesenchymal stromal cells found in the perivascular space of adipose tissue. ADSCs have the advantage of abundance and easy access when compared with other stem cell types [6]. ADSCs express common stem cell surface markers, genes, and differentiation potentials as MSCs [7]. ADSCs have demonstrated efficacy in experimental studies of urologic conditions [8, 9].

SkMSCs are used mainly in artificial injured model including pelvic nerve injury [10, 11]. As a stem cell source of autologous transplantation, SkMSCs have several advantages because skeletal muscle is the largest organ in the body and can be obtained relatively easily and safely. One of other advantages of SkMSCs is that they can be harvested easily during surgery. 

Cells in the CD34^+^/CD45^−^ fraction (Sk-34 cells) and CD3^−^/CD45^−^ fraction (Sk-DN cells) are able to synchronously reconstitute nerve-muscle blood vessel units after transplantation. Transplantation of SkMSCs causes significant functional recovery through cellular differentiation into skeletal muscle cells, vascular cells (vascular smooth muscle cells, pericytes, and endothelial cells), and peripheral nervous cells (Schwann cells and perineurium) [12, 13].

## 3. Mechanisms of Stem Cell in Recovery of Bladder Dysfunction

Stem cells (SCs) are self-renewing adult stem cells with multipotent differentiation potential. SCs can become many types of tissues either via transdifferentiation or via cell fusion and allow the regeneration and functional restoration [14] and are an important source for cell replacement [15]. They can serve as vehicles for gene transfer, proliferate, and differentiate into bladder smooth muscle cells to repopulate damaged bladder.

### 3.1. Migration

Recruitment of SCs to the bladder in BOO appears to be associated with increased blood flow and decreased tissue hypoxia, which contributes to improvement in histopathological and functional parameters [16].

MSCs are recruited to inflammation, ischemia, or damaged sites in response to specific chemokines expressed by damaged tissue [17]. A large body of literature exists in the roles of by recruited stem cells in a number of different organ systems and also it is demonstrated in injured bladder, too [18–21]. 

### 3.2. Differentiation

The ideal mechanism of stem cell therapy is differentiation, but only a few studies have demonstrated real differentiation into bladder smooth muscle (Table 1). Bladder regeneration by differentiation has been frequently reported in nonpathogenic bladder model. Differentiation pathway plays at most a minor role in the therapeutic effect by SCs transplantation. One explanation is the immortal strand hypothesis [22]. Labeled DNA in dividing cells will be quickly diluted by cell divisions but will be retained for much longer periods in slowly dividing stem cells. If the segregation of sister chromatids into stem cell daughters is not random, and if the stem cell retains the older unlabeled template strands, then the stem cell will lose all labels by the second division after administration of the label as a pulse. 

Successful differentiation of stem cells into smooth muscle for bladder repair and replacement was reported by several studies in non-pathogenic model for tissue regeneration [23, 24].

### 3.3. Paracrine Effect

While differentiation has long been considered the main mechanism, it is logical to hypothesize that paracrine release of cytokines and growth factors by transplanted MSCs or their neighboring cells is responsible for the observed effects.

In this regard, SCs secretory factors have been shown to exert therapeutic effects by the modulation of local and systematic inflammatory responses, the stimulation of local tissue regeneration, and/or recruitment of host cells. MSCs themselves could not substitute the damaged cells directly but secrete a growth factor and contribute to reducing fibrosis through paracrine mechanisms [25].

 BM-MSCs or ADSCs could secrete many growth factors including hepatic growth factor (HGF), nerve growth factor (NGF), brain-derived growth factor (BDNF), glial-derived growth factor (GDNF), insulin-like growth factor (IGF), vascular endothelial growth factor (VEGF), and ciliary neurotrophic growth factor (CNTF) [26] and play an essential part in the antifibrosis effects in injured organ, which implies that reducing fibrosis is managed by paracrine mechanisms rather than by cell incorporation [27–29]. Among the growth factors, HGF is a potent mitogen for hepatocytes, is secreted by MSCs, plays an essential part in the angiogenesis and regeneration of the tissue, and acts as a potent antifibrotic agent [30, 31].

 In addition to antifibrotic mechanisms, BM-MSCs or ADSCs may provide antioxidant chemicals, free radical scavengers and heat shock proteins in ischemic tissue [32].

## 4. Stem Cell Therapy in Pathologic Model of Bladder Dysfunction

 To date, the BOO model is the prominent model for bladder dysfunction, and other several pathologic models are in challenging state. The BOO model and cryo-injured model have a similar mechanism to induce bladder dysfunction. The BOO model also has ischemia mechanism which is similar to ischemia model. To date, for spinal cord injuries, there have been no studies published describing the grafting of stem cell into the injured bladder. Most studies have dealt with directly grafting of stem cells or bone marrow derived cells into injured spinal cord directly.

### 4.1. Bladder Outlet Model

Bladder outlet obstruction (BOO) caused by collagen deposit is one of the most common problems in elderly males. The collagen deposition in bladder occurs frequently during development of various pathological processes and eventually cumulates in bladder fibrosis, and finally induces a flaccid bladder. This bladder fibrosis adversely affects the smooth muscle function and the bladder compliance [33]. Bladder dysfunction after BOO is related to alterations in ultrastructure properties of the smooth muscle and collagen. In bladder outlet obstruction, bladder instability was found [34]. Compensated bladder dysfunction with overactive bladder is expected after 6 weeks [33]. 

Recently, Lee et al. have reported that transplantation of primary human MSCs labeled with nanoparticles containing superparamagnetic iron oxide into the bladder wall of a rat BOO model inhibited bladder fibrosis and induced improvement of bladder dysfunction [19]. 

Growth factors have been reported in the bladder development and the remodeling of the bladder wall after outlet obstruction [35]. This finding was also demonstrated by Song et al. [20] that displayed human MSCs overexpressing HGF by pairing clonal human MSCS with HGF inhibited collagen deposition and improved cystometric parameters in BOO of rats.

Woo et al. [21] reported that MSCs engraftment had improved compliance compared to those without engraftment. Polymerase chain reaction revealed a 2-fold increase in CCL2 expression, but there were no significant changes in other chemokine. Additionally, CCL2 in the BOO model was identified by Tanaka et al. [36]. In their study, bone marrow derived cells were present in the urothelial and stromal layers after BOO. An activated epidermal growth factor receptor was found in cells associated with bone marrow derived cells [36].

A possible explanation for bladder dysfunction is decreased local blood flow, which means significant tissue ischemia. Increased intraluminal pressure is thought to cause vessel compression, which is further aggravated by fibrosis and hypertrophy [37, 38].

 Not all the BOO model results reveal overactivity. Nishijima et al. [39] showed that transplanted bone marrow cells may improve bladder contractility by differentiating into smooth muscle-like cells in underactive bladder by BOO.

The applicability of MSCs to the treatment and/or prevention of bladder dysfunction by BOO would provide a new, potentially powerful addition to the limited armamentarium of existing therapies.

### 4.2. Bladder Ischemia Model

The bladder ischemia model is established using bilateral iliac artery ligation [40] or hyperlipidemia [41]. Several studies [42] showed that artery stenosis and blood insufficiency can cause significant changes of the bladder's structure and functionality. The mechanism of ischemia-induced bladder dysfunction is complicated, which may be related to ischemic denervation. This causes M-cholinergic receptor hypersensitivity to acetylcholine [43] and results in detrusor overactivity which leads to a more ischemic state of bladder wall. Considering the high rate of ischemic changes in the elderly, it is possible that the ischemia rat model could also be used for bladder dysfunction which is caused by aging detrusor.

Chen et al. [40] reported that pathological and functional changes of the bladder ischemic model are similar to the human aging detrusor. Chen et al. [40] also reported that the injection of stem cell suspension into the common iliac artery in rats with ischemic bladder, followed by intragastric administration of doxazosin mesylate, which makes transplanted stem cells regenerate in the bladder tissue, increases the percentage of smooth muscle content and nerve cells, and improves bladder detrusor function. Huang et al. [41] showed that direct injection to the bladder or intravenous injection of ADSCs improved urodynamics and tissue parameters in the rat model of hyperlipidemia associated overactive bladder.

 Azadzoi et al. [44] found that hyperlipidemia induced chronic ischemia increases transforming growth factor-*β*1 in the bladder which leads to fibrosis and noncompliance. 

### 4.3. Diabetes Model

Daneshgari et al. [45] proposed that diabetic bladder dysfunction (DBD) typically evolves in a time-dependent progression of both storage and voiding problems. The early phase of DBD manifests as detrusor overactivity, which represents leading to urinary frequency and urgency. However, over time, progressive oxidative stress and neuropathy lead to decompensation of the detrusor musculature, thereby leading to the underactive or atonic bladder. 

Zhang et al. [46] reported that improved voiding function was noted in ADSCs-treated rats as compared with phosphate-buffered saline-treated rats. DBD pattern was hypocontractile bladders in their experimental model. Though some ADSCs differentiated into smooth muscle cells, paracrine pathway seems to play a main role in this process as well. This means that transplantation of ADSCs could result in reduction of apoptosis and preservation of “suburothelial capillaries network.” 

### 4.4. Spinal Cord Injured Model

Spinal cord injury (SCI) induces complete deterioration of bladder compliance, function, infection, and other lower urinary tract complications [47]. SCI rat exhibited increased bladder wall thickness that contained a larger percentage of collagen [48]. The goal of bladder treatment in patients with SCI is to reduce infections, preserve renal function, and to improve patient' quality of life. Transplantation of neural stem cells into the injured spinal cord has been reported to improve bladder function in animal models [49]. However, no study has shown whether MSCs grafting into the bladder wall can influence bladder function following SCI. 

 As the mature central nervous system cannot generate new neurons and glial cells, bladder functional recovery is limited following SCI. However, recent studies suggest that transplanted neural progenitor cells promote recovery of the bladder function through regeneration of the injured site [49–52]. In most of these studies, stem cells have been injected into the injured lesion directly with a needle. Hu et al. [53] showed that intravenously transplanted bone marrow stromal cells (BMSCs) survived in the L3-4 and had beneficial effects on the recovery of bladder function in the rats after spinal cord injury.

### 4.5. Cryo-Injured Model

The cyro-injured model induces bladder hypertrophy with loss of smooth muscle and increase of collagen which represent a similar mechanism with the BOO model [54]. The major outcome of the stem cell transplantation in the cryo-injured bladder seems to be that of preventing the increase in size of surviving smooth muscle cells (SMCs) along with a poor differentiation drive to SMC lineage. This can exert an important effect on the remodeling process in the injured bladder, which is characterized by the development of a compensatory SMC hypertrophy. As reported by Somogyi et al. [55], functional (and structural) impairing of innervation (and reinnervation) in the cryo-injured bladder can induce long-lasting tonic contractions to more effectively empty the bladder. 

 De Coppi et al. [54] showed protective effect of MSCs transplantation using AF-MSC and BM-MSC. They concluded that stem cell transplantation has a limited effect on smooth muscle cell regeneration. Instead, it can regulate postinjury bladder remodeling, possibly via a paracrine mechanism.

 Huard et al. [56] showed that injected muscle-derived cells (MDCs) are capable of not only surviving in the lower urinary tract, but also improving the contractility of the bladder in cryo-injured model. They used modified MDCs which were genetically engineered to express the gene encoding *β*-galactosidase.

 Sakuma et al. [57] showed that dedifferentiated fat cells can differentiate into smooth muscle cell lineages and contribute to the regeneration of bladder smooth muscle tissue using human adipocyte derived dedifferentiated fat cells in cryo-injured model. 

### 4.6. Other Bladder Dysfunction Model

Nitta et al. [10] showed that significantly higher functional recovery was noted by transplantation of skeletal muscle-derived multipotent stem cells in bladder branch of the pelvic plexus (BBBP) injured model. The transplanted cells showed incorporation into the damaged peripheral nerves and blood vessels after differentiation into Schwann cells, perineurial cells, vascular smooth muscle cells, pericytes, and fibroblasts around the bladder.

 Kwon et al. [11] reported that similar cell transplantation of muscle-derived cells isolated was achieved using the preplate technique, in the unilaterally transected pelvic nerve model in rats. They performed functional measurement of intravesical pressures by electrical stimulation of the transected pelvic nerve and obtained significant functional recovery through crosssectional group comparison analysis 2 weeks after transplantation.

### 4.7. Bladder Regeneration in Nonpathogenic Model

For bladder tissue engineering, three pioneering studies have demonstrated that embryoid body derived stem cells or BMSCs seeded on small intestinal submucosa (SIS) facilitated the regeneration of partially cystectomized bladder [58–60]. Recently hair stem cells and ADSCs seeded on bladder acellular matrix (BAM) have also shown bladder regeneration potential [61, 62]. In studies which deal with the use of synthetic scaffolds instead of SIS and BAM, Sharma et al. reported that BMSCs seeded on poly (1,8-octanediol-co-citrate) thin film supported partial bladder regeneration [63], and Tian et al. showed that myogenic differentiated BMSCs seeded on poly-l-lactic acid scaffold exhibited bladder engineering potential [64, 65]. Likewise, poly-lactic-glycolic acid seeded with myogenically differentiated human ADSCs maintained bladder capacity and compliance when grafted in hemicystectomized rats [66].

Bladder tissue engineering using MSCs might show better results than by using differentiated cells. MSCs were shown to migrate to the bladder grafts and differentiate into SMC [67]. These cells achieved fast replacement of the grafts with appropriate neural function and less fibrosis [57].

### 4.8. Human Study

The human bladder is one organ to which stem cell technology could be applied. But human studies are scarce and there were no studies regarding pathologic bladder dysfunction. Studies could only be found in regards to the neobladder and urethral sphincter. Urologists need an appropriate substitute for the traditional conduits and neobladders, given their complications of adhesions, mucus formation, incomplete emptying, and metabolic and malignant transformations. Pioneering research has created artificially engineered bladder tissues using autotansplantation [68]. Urothelial and muscle cells obtained by bladder biopsy were grown in a culture for 7 weeks and transplanted in layers on a biodegradable bladder-shaped scaffold made of collagen and polyglycolic acid. During a mean follow-up of just under 4 years, all patients had improved bladder function with no major surgical or metabolic complications.

## 5. Discussion

### 5.1. Stem Cell Selection

Classification into embryonic or adult stem cells is useful to distinguish ethical issues associated with the destruction of an embryo to yield cells for research purposes. Whereas embryonic stem cells are considered to be pluripotent, adult stem cells are thought to be restricted in their potency by their tissue of origin. This concept remains controversial, as adult stem cells can also produce mature cells not normally seen in their tissue of origin under certain conditions. The exact molecular mechanism of this phenomenon, known as *plasticity* or *transdifferentiation,* is yet to be discovered [69]. However, the recent discovery of induced pluripotent stem cells (IPSs) has shown some light into the molecular basis of stem cells [70]. These IPSs were mostly derived from skin, but recently urinary tract derived IPSs were shown to be more efficient than skin-derived iPSCs in bladder differentiation which was demonstrated by expression of urothelial-specific markers including uroplakins, claudins, and cytokeratin and stromal smooth muscle markers including *α*-smooth-muscle actin, calponin, and desmin. These disparities highlight the epigenetic differences between individual IPS lines and represent the importance of organ-specific IPSCs for tissue-specific studies [71].

Although immature (embryonic or fetal) stem cells may be more efficient than adult counterparts in points of providing the supporting acellular matrix with multipotent progenitor cells which could differentiate into distinct cell lineages, fetal-type MSCs were not superior to adult-type MSCs in terms of contributing to the formation of new differentiated SMCs or vascular cells despite the nominal higher plasticity of immature stem cells in real experiments [54, 72].

### 5.2. Tumorigenesis

Regardless of their tissue origin, all stem cell types could not avoid the main issue of tumorigenesis and if they themselves become tumors or whether they encourage the growth/metastasis of existing tumors. While several studies on the former aspect have generated results due to tumor cell line contamination, an ever increasing number of publications are sounding the alarm on the latter issue. However, these studies relied on the use of animal models transplanted with tumor cell lines, an approach whose clinical relevance has long been questioned. This is also the main factor as to the main reason why human study is not easy. 

Clinical human studies of muscle-derived cell transplantation have recently been performed in only urethral sphincter dysfunction, and no studies have been performed yet in bladder dysfunction. Eight women with stress urinary incontinence (SUI) were treated with muscle-derived stem cells [73–75]. 

Although, autologous transplantation of bladder stem cells could be possible and could avoid the limitation of theoretical and ethical standpoints, the exact culture conditions to direct autologous nonbladder stem cells to transdifferentiate into urothelial cells are yet to be established.

### 5.3. Route of Transplantation

In contrast to local injection of MSCs, intravenously administered MSCs are distributed throughout the whole animal. Furthermore, there is the concern of causing capillary clogging when larger cell types such as MSCs are infused, a complication that could result in hemodynamic compromise, interference with pulmonary gas exchange, and respiratory distress [76]. Intravenously injected MSCs are localized mainly to the pulmonary capillary bed [77]. Local injection may have a better effect than intravenous injection does. A recent study by Huang et al. [41] also showed better improvement of bladder function in the direct injection group than in the systemic injection group. 

### 5.4. Metabolic Memory

For administration of SC, “metabolic memory” has to be considered first. It suggests that SCs derived from pathologic animals such as diabetes behave differently than those derived from healthy animals [78]. Be that as it may, it seems most likely that only individuals with a metabolic derangement leading to tissue damage will seek out this form of treatment. Hence, the diabetic or other systemically ill animal models are appropriate research subjects for preclinical research.

### 5.5. Gene Therapy

Although gene therapy to be efficacious and one of the promising therapeutic options, effective gene transfer into stem cells must be achieved without inducing detrimental effects on their biological properties. Although modification of MSCs to overexpress HGF has an effective means to maintain or enhance the capacity of MSCs and to be efficacious for bladder fibrosis therapy [20], the choice of vector for cell transduction should be carefully considered. The selected vector should have high transduction efficiency and should ensure stable and long-term transgene expression from the cell vehicle and be devoid of any damaging effect on cell viability.

### 5.6. Limitation of Experimental Study

Without intermediary examination, such as survival urodynamic or bladder biopsy at a midpoint in the experiment, also, whether stem cells may serve a more preventative or ameliorative role are too early to determine. It is hard to know whether obstructed bladders appear to undergo early signs of obstruction that are then later reversed or the pathological process is avoided altogether.

## 6. Conclusions

There are interesting results with experimental use of stem cells to treat bladder dysfunction. The use of MSCs has shown great promise in several animal studies.

Although significant challenges are still need to overcome challenges for human application, this novel technology has the potential to become a major source of cells for treatment of bladder dysfunction. In order to determine the exact role of stem cells in treatment of bladder dysfunction, more trials are needed.

## Figures and Tables

**Table 1:** Studies about stem cell therapy for bladder dysfunction.

Source	Mechanism of bladder dysfunction	Stem cell	Animal	Transplantation route	Tracking of stem cell	Bladder activity	Functional study	Smooth muscle differentiation	Remarks
Lee et al. [19]	BOO	Human BM-MSC	Rat	Direct transplantation into bladder	Iron oxide nanoparticle	Overactivity	UDS done: improvement of ICI, MVP, and RU	No	In vivo MRI examination for tracking MSC

Song et al. [20]	BOO	Human BM-MSC	Rat	Direct transplantation into bladder	None	Overactivity	UDS done: improvement of ICI and MVP	Yes	Transfection of human HGF gene into MSC

Woo et al. [21]	BOO	Mice BM-MSC	Mice	Intravenous injection	None	Overactivity	UDS done: improvement of capacity and compliance	No	Expression of chemokine CCL2

Tanaka et al. [36]	BOO	BMD cell	Mice	Direct transplantation into BM	GFP labeling	Overactivity	UDS done: improvement of compliance and capacity	No	BOO was made by periurethral collagen injection, radiation was induced, and CCL2 induction increased.

Nishijima et al. [39]	BOO	BM cell	Rat	Direct transplantation into bladder	GFP labeling	Underactivity	UDS done: improvement of ICI and RU	Yes	Underactive model was created

Chen et al. [40]	Chronic ischemia by bilateral iliac artery ligation	BM-MSC	Rat	Intra-arterial injection	BrdU labeling	Underactivity	UDS done: improvement of ICI	No	Doxazosin mesylate was intragastrically administrated

Huang et al. [41]	Chronic ischemia by hyperlipidemia	ADSC	Rat	Direct transplantation into bladder/intravenous injection	EdU labeling	Overactivity	UDS done: improvement of ICI and VV	Yes	Direct transplanted group showed better differentiation and better functional improvement

Zhang et al. [46]	Diabetes	ADSC	Rat	Direct transplantation into bladder/intravenous injection	EdU labeling	Overactivity. underactivity	UDS done: improvement of ICI and VV	No	Direct transplanted group showed better differentiation and better functional improvement

De Coppi et al. [54]	Cryoinjured	BM-MSC and AF-MSC	Rat	Direct transplantation into bladder	GFP labeling	N/A	N/A	Yes	Cell fusion in vitro

Huard et al. [56]	Cryoinjured	Muscle-derived cell	Mice	Direct transplantation into bladder	LacZ staining	Underactivity	Contractility test using muscle strip done: improvement of contractility	Yes	Ex vivo gene transfer using *β*-galactosidase

Sakuma et al. [57]	Cryoinjured	Adipocyte derived fat cell	Mice	Direct transplantation into bladder	GFP labeling	Underactivity	N/A	Yes	TGF-*β* signaling for smooth muscle differentiation

Nitta et al. [10]	Pelvic nerve injured	Sk-MSC	Rat	Damaged nerve lesion	GFP labeling	Underactivity	UDS done under electrical stimulation: improvement of IVP	Yes	Autologous and heterologous models were experimented together

Kwon et al. [11]	Pelvic nerve injured	Muscle-derived cell	Rat	Damaged nerve lesion	Enkephalin staining	Underactivity	UDS done under electrical stimulation: improvement of IVP	No	Autograft

Mitsui et al. [49]	SCI	Neural stem cell	Rat	Damaged cord lesion	BrdU labeling	Overactivity	UDS done: improvement of VV and VP	No	No difference in the incidence of detrusor overactivity

Temeltas et al. [52]	SCI	Bone marrow stromal cell	Rat	Damaged cord lesion	E-NACM staining	Overactivity	UDS done: improvement of VV, RU and VP	N/A	Glial restricted precursor was also treated

Hu et al. [53]	SCI	Bone marrow stromal cell	Rat	Intravenous injection	BrdU labeling	Overactivity	UDS done: improvement of compliance and capacity	N/A	External urethral sphincter activity was checked

BOO: bladder outlet obstruction; SCI: spinal cord injury; MSC: mesenchymal stem cell; BMD: bone marrow derived cell; ADSC: adipose derived stem cell; AF-MSC: amniotic fluid derived mesenchymal stem cell; Sk-MSC: Skeletal muscle derived mesenchymal stem cell; UDS: urodynamic study; ICI: inter-contraction interval; MVP: maximal voiding pressure; RU: residual urine; VV: voided volume; VP: voiding pressure; IVP: intravesical pressure; GFP: green fluorescent protein.
